# The Claybury Asylum

**Published:** 1893-06-24

**Authors:** 


### THE CLAYBURY ASYLUM.

Saturday last witnessed the formal opening of the new Asylum at Claybury, near Woodford, Essex, a large number of people being present. The ceremony took place in the Recreation Hall, Mr. Strong, chairman of the Asylums Committee, in the chair, supported by many members of the County Council. In opening the proceedings, Mr. Strong said that this building was inaugurated in order that those inhabitants of London whose mental condition necessitated their being placed under restraint might be well and carefully looked after. As yet the buildings were somewhat bare, but these were to be papered, painted, and decorated, and made as bright as possible with flowers and plants, so that the inmates might have as many interests as possible. Since these buildings were begun the number of lunatics to be provided for had increased, and it was now found that the accommodation would be insufficient, and it would be necessary to look for a new site.

After a few words from Mr. Martineau, the chairman of the Claybury Sub-Committee, all present proceeded to the main entrance, which was then formally opened by him with a key presented by Mr. Hine, the architect.

The estate on which the asylum stands covers some 269 acres of ground, the buildings themselves extending over about 20 acres. The asylum is situated at the highest point, the windows commanding wide views on all sides, especially over Epping Forest. It is built in red brick, the ground plan being of irregular shape, narrowing towards the frontage. It is designed on the block system, one-storey corridors connecting the various blocks, and communicating with the administrative department, which occupies the central portion. Provision is made for 2,000 patients—800 males and 1,200 females—there being also accommodation for between 200 and 300 officers and attendants. There are separate blocks for sick, infirm, and recent cases, epileptics, acute cases, chronic cases, and the laundry. Workshops for various handicrafts are provided for the male patients, and a large needle-room for female patients. Every ward has its bath-room, besides general bath-rooms. The asylum is entirely lighted by electricity, and heated throughout by the "Plenum" system, all steam pipes, &c., being in the basement storey, out of the reach of the patients. Some idea may be gained of the enormous size of the buildings by mention of the fact that it contains 4,700 windows and 2,600 doors. Glazed brickwork and tiles have been used in the corridors, the rooms having varnished wooden dados. The chapel will seat 800 people, and the Recreation Hall, which is a splendid room, 120 feet in length, with pitch pine fittings and polished floor, has seating capacity for 1,000 persons. In the windows in the Recreation Hall may be seen, in stained glass, the armorial bearings of constituencies returning members to the County Council. The sewage arrangements appear to be designed on the best modern principles.

A fully detailed plan of the buildings is given in Vol. II. of "Hospitals and Asylums of the World."

In the event of an outbreak of infectious disease, an isolation hospital has been provided with accommodation for 20 patients.

